# 

**Published:** 1883-03

**Authors:** 


					﻿VOL. III.
MARCH, 1883.
NO. 3.
THE
HOMEOPATHIC PHYSICIAN,
A MONTHLY JOURNAL OF MEDICAL SCIENCE.
“Magna est veritas, el praevalebit.'"
HL CT. LHJH3, Js/L. ZD.,
EDITOR.
CONTENTS:
£See inside of Cover.)
PHILADELPHIA:
No. 2109 CHESTNirr STREET.
JTH-W YOBK:
A. T>. CHATTERTON, PUBLISHING COMPANY^ PUBLISHER’S AGENTS.
X O 1ST ID O 3<T :
ALFRED HEATH & CO,, Sole Agents eob Great Britain.
114 Ebury Street,. Eaton Square. ,
Yearly Subscription, $2.50, invariably in advance,.
Entered at the Philadelphia Post-Office as Second- Class Mail Matter.
				

